# Genome organization across scales: mechanistic insights from *in vitro* reconstitution studies

**DOI:** 10.1042/BST20230883

**Published:** 2024-03-07

**Authors:** Elisa Oberbeckmann, A. Marieke Oudelaar

**Affiliations:** 1 Department of Molecular Biology, Max Planck Institute for Multidisciplinary Sciences, Am Fassberg 11, 37077 Göttingen, Germany; 2 Genome Organization and Regulation, Max Planck Institute for Multidisciplinary Sciences, Am Fassberg 11, 37077 Göttingen, Germany

**Keywords:** chromatin, 3D genome organization, *in vitro* reconstitution, loop extrusion, nucleosome

## Abstract

Eukaryotic genomes are compacted and organized into distinct three-dimensional (3D) structures, which range from small-scale nucleosome arrays to large-scale chromatin domains. These chromatin structures play an important role in the regulation of transcription and other nuclear processes. The molecular mechanisms that drive the formation of chromatin structures across scales and the relationship between chromatin structure and function remain incompletely understood. Because the processes involved are complex and interconnected, it is often challenging to dissect the underlying principles in the nuclear environment. Therefore, *in vitro* reconstitution systems provide a valuable approach to gain insight into the molecular mechanisms by which chromatin structures are formed and to determine the cause-consequence relationships between the processes involved. In this review, we give an overview of *in vitro* approaches that have been used to study chromatin structures across scales and how they have increased our understanding of the formation and function of these structures. We start by discussing *in vitro* studies that have given insight into the mechanisms of nucleosome positioning. Next, we discuss recent efforts to reconstitute larger-scale chromatin domains and loops and the resulting insights into the principles of genome organization. We conclude with an outlook on potential future applications of chromatin reconstitution systems and how they may contribute to answering open questions concerning chromatin architecture.

## Introduction

Eukaryotic genomes are folded into distinct 3D structures across different scales. The resulting packaging of the long and stiff DNA molecules allow them to be compacted into the finite space of micron-sized nuclei. In addition, the spatial organization of DNA is thought to have a key regulatory function in many nuclear processes, including transcription, replication, DNA repair, and chromosome segregation. At the smallest scale, DNA is organized into nucleosome core particles, which consist of 147 base pairs (bp) of DNA wrapped around a histone octamer [1–3]. Connected by short DNA linkers, nucleosome core particles form nucleosome arrays, which are further assembled into heterogenous chromatin fibers with diameters of ∼8–24 nm [4–6]. Nucleosomes play an important role in the regulation of transcription [7]. In addition, wrapping of the negatively charged DNA around the positively charged histone octamers (partially) neutralizes the negative charge of DNA molecules and facilitates their bending; the organization of the genome into chromatin therefore contributes a moderate level of compaction [8].

Different types of chromatin are spatially separated in the nucleus, resulting in the formation of functionally distinct compartments. The A compartment consists of euchromatin, which is characterized by the presence of active genes and histone modifications and a relatively low level of compaction. Regions of heterochromatin, which are generally transcriptionally silent, bear inactive histone modifications, and have a more compact conformation, constitute the B compartment. Compartmentalization of euchromatin and heterochromatin is thought to be dependent on phase separation, driven by molecular affinities between the chromatin factors that associate with the distinct chromatin types [9].

An additional organizing principle of eukaryotic genomes is loop extrusion, which results in the formation of distinct 3D structures throughout the cell cycle [10–12]. Loop extrusion is mediated by structural maintenance of chromosomes (SMC) complexes, which are multi-subunit, ATP-dependent motor proteins that translocate along chromatin and thereby extrude progressively larger loops [13]. Eukaryotes have three main classes of SMC complexes with distinct functions, which include condensin, cohesin and SMC5/6 [14]. Condensin mediates the compaction and segregation of mitotic chromosomes during cell division. Cohesin mediates sister chromatid cohesion during mitosis and regulates the organization of (mammalian) interphase genomes into topologically associating domains (TADs), which are thought to play an important role in gene regulation. SMC5/6 has been implicated in DNA damage repair and replication [14]. Non-canonical SMC proteins (for example, SMCHD1 [15]) and non-SMC proteins (for example, Polycomb-group proteins [16], the Mediator complex [17], and YY1 [18]) also have a role in the regulation of chromatin architecture during interphase.

Despite the importance of the 3D organization of the genome for several fundamental nuclear processes, our understanding of the underlying molecular mechanisms remains incomplete. A major hurdle to identifying the detailed mechanisms involved is the complexity of the crowded nuclear environment, in which dynamic processes are difficult to visualize and the primary functions of key proteins and their interacting partners are difficult to dissect due to the cascade of (secondary) effects triggered by their perturbation. These limitations can be addressed by the use of reconstitution approaches, which allow researchers to study proteins and processes of interest outside the complex cellular milieu and thus to gain more direct insight into molecular mechanisms and cause-consequence relationships. In this review, we discuss recent progress in the *in vitro* reconstitution of genome structures across scales and the insights into the principles of genome organization that have emerged. We first focus on the reconstitution of nucleosome fibers and the resulting insights into the mechanisms that determine nucleosome positioning. Next, we discuss recent efforts to reconstitute larger-scale 3D chromatin structures and loop extrusion by SMC complexes, and how these experiments have contributed to our understanding of the underlying molecular mechanisms. We conclude by highlighting important open questions in the field that could be addressed by *in vitro* reconstitution approaches in the future.

## Reconstitution of nucleosome positioning

**Figure 1.**
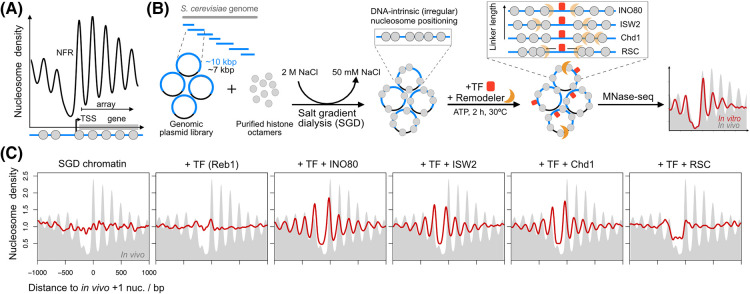
*In vitro* reconstitution of nucleosome positioning in *S. cerevisiae*. (**A**) Stereotypical nucleosome-free region (NFR)-array pattern at transcription start sites (TSSs) in wild-type *S. cerevisiae* chromatin. Gray circles indicate nucleosomes. Nucleosome density is derived from micrococcal nuclease-sequencing (MNase-seq) data [23] and averaged over all TSSs. For MNase-seq, chromatin is digested with the endo- and exonuclease MNase, which predominantly cleaves nucleosome-free DNA. The protected, nucleosomal DNA is then purified and sequenced to infer the nucleosome positions. (**B**) Schematic overview of genome-wide *in vitro* reconstitution experiments to study nucleosome positioning in *S. cerevisiae* based on salt gradient dialysis (SGD). Nucleosomes are assembled by incubating purified histone octamers with a DNA template in a high-salt buffer, allowing for spontaneous assembly of nucleosomes as the salt is slowly dialyzed away. The positioning of nucleosomes in the SGD chromatin is DNA-intrinsic and irregular. By incubating SGD chromatin with a transcription factor (TF), ATP-dependent chromatin remodeler and ATP, regular nucleosome positioning patterns can be reconstituted and analyzed by MNase-seq. (**C**) Example of MNase-seq data derived from the reconstitution approach described in panel B (red line) [24]. SGD chromatin was prepared with recombinant yeast histone octamers at a high nucleosome density (histone-to-DNA ratio = 0.8) and incubated with the TF Reb1 and/or the indicated remodelers, leading to the formation of distinct nucleosome density profiles. The MNase-seq data are averaged over Reb1-bound TSSs. Comparison with the *in vivo* MNase-seq data (gray background) highlights differences in NFR width and nucleosome spacing between the different *in vitro* conditions.

The first genome-wide nucleosome maps that were generated using micrococcal nuclease-sequencing (MNase-seq) in the early 2000s revealed that nucleosomes are not randomly distributed across the genome, but form a stereotypical pattern at actively transcribed genes [19–22]. This pattern is characterized by a nucleosome-free region (NFR) at transcription start sites (TSSs), which is flanked by regularly spaced and phased nucleosome arrays over the gene bodies (Figure 1A). Although this pattern was identified across eukaryotic organisms [19–22], the underlying mechanisms and the extent to which nucleosome positioning is encoded by the DNA sequence *in cis* or dependent on the action of ATP-dependent chromatin remodelers *in trans* [25–27] remained unclear. The *cis* regulation model proposes that the strength of the interactions between DNA and histones is the main driver of nucleosome positioning and that the function of remodelers is restricted to mobilizing nucleosomes without determining their destination [28–30]. This model is based on the fact that eukaryotic genomes contain sequences with high and low affinity for nucleosomes, which depend on the biophysical properties of DNA. This results in a genomic code that can predict nucleosome positioning. In contrast, the *trans* regulation model proposes that nucleosomes are predominantly positioned by *trans*-acting factors, including chromatin remodelers, sequence-specific transcription factors (TFs), and the transcription machinery, which can overrule the inherent nucleosome affinity of the DNA sequence [31–34].

Over the last decade, innovative *in vitro* experiments have revealed that the combined action of *trans*-acting remodelers and TFs is the main driver of nucleosome positioning. This has been most clearly demonstrated in studies based on reconstitution of yeast chromatin with the salt gradient dialysis (SGD) method (Figure 1B) or histone chaperone-based chromatin assembly systems. In combination with a genome-wide plasmid library or genomic DNA as template, these approaches enable analysis of nucleosome positioning patterns across the genome. Notably, reconstituted SGD chromatin lacks the characteristic NFR-array pattern that is observed surrounding TSSs *in vivo*, which indicates that nucleosome positioning is not encoded by DNA sequence (Figure 1C) [33]. However, the incubation of SGD chromatin with yeast whole-cell extract and ATP does result in a typical *in vivo*-like nucleosome positioning pattern [35]. These observations therefore clearly demonstrate that the NFR-array pattern is generated by an active, ATP-dependent mechanism.

In addition to their contribution to the debate about *cis* and *trans* regulation of nucleosome positioning, SGD reconstitution studies have provided insight into the function and mechanisms of remodelers, which are difficult to study *in vivo* due to their redundancy [36,37]. Interestingly, experiments based on incubation of SGD-reconstituted chromatin with purified remodelers and TFs have revealed that different remodelers have distinct mechanisms of action. For example, INO80, RSC and Chd1 can process nucleosome-positioning signals in the DNA sequence, whereas ISWI remodelers exclusively co-operate with TFs to position nucleosomes [38–40]. Similar *in vitro* reconstitution experiments have been used to study how nucleosome density influences the nucleosome positioning patterns that are generated by remodelers. This question is difficult to address *in vivo*, as it is not straightforward to reduce nucleosome density due to the presence of multiple copies of histone genes and the strong, often lethal, phenotypes of their perturbation [41]. However, *in vitro*, nucleosome density can easily be modified by adapting the histone-to-DNA ratio during SGD reconstitution. This approach has demonstrated that ISWI remodelers, INO80 and Chd1 contain dedicated subunits or domains that function as a ‘ruler’ and set regular distances between two nucleosomes (Figure 1B,C) [23]. In some cases, the distance set by the remodeler depends on the nucleosome density. For example, the ruler of INO80 can adapt and set longer distances between nucleosomes at lower nucleosome densities compared with higher densities. In contrast, Chd1 always sets very short distances, regardless of nucleosome density [23].

In addition to remodelers and sequence-specific TFs, it has been proposed that the process of active transcription, for example by RNA polymerase II, also influences nucleosome positioning [33]. Since the stereotypical NFR-array pattern is mainly found at actively transcribed genes, it initially remained unclear whether this pattern serves as a prerequisite for active transcription, or is formed as a result of active transcription. *In vitro* reconstitution experiments in a transcription-free system provided helpful insight into this debate, as they demonstrated that TFs and remodelers are sufficient to generate *in vivo-*like NFR-array patterns [23,39]. Importantly, this has been confirmed by *in vivo* experiments based on a rapid anchor-away system in *Saccharomyces cerevisiae* coupled with the knock-out of multiple remodelers [42]. Although transcription is not necessary for nucleosome positioning, it is important to note that it may still have an important (indirect) role in nucleosome positioning *in vivo*, by recruiting remodelers to chromatin. For example, it has been shown that Chd1 interacts with the histone chaperone FACT, which is required for efficient transcription elongation [43,44]. This indicates that active elongation might promote the recruitment of remodelers to chromatin.

## Reconstitution of higher-order chromatin structures

Over the last two decades, it has become clear that chromatin is further organized into distinct higher-order structures, including self-interacting chromatin domains that span a wide range of sizes [45]. Based on the observations that chromatin domains correlate with distinct chromatin states and that domain boundaries frequently overlap with active gene promoters, a connection between higher-order genome organization and transcription has been proposed [46,47]. However, the cause-consequence relationship remained unclear, and it was not understood whether transcription drives the formation of chromatin domains or whether chromatin domains influence transcription patterns. Furthermore, despite the relatively well-documented importance of loop extrusion in mammalian genome organization [48,49], it remained unclear to what extent a driving role for loop extrusion in the organization of interphase genomes is conserved across eukaryotes. Due to the complex interplay between loop extrusion, transcription, chromatin state, and chromatin domains [50–52], it is challenging to dissect the contribution of these processes to genome organization *in vivo*. *In vitro* reconstitution systems therefore provide a useful approach to help determining the conserved, core principles that drive higher-order genome organization in eukaryotes.

**Figure 2.**
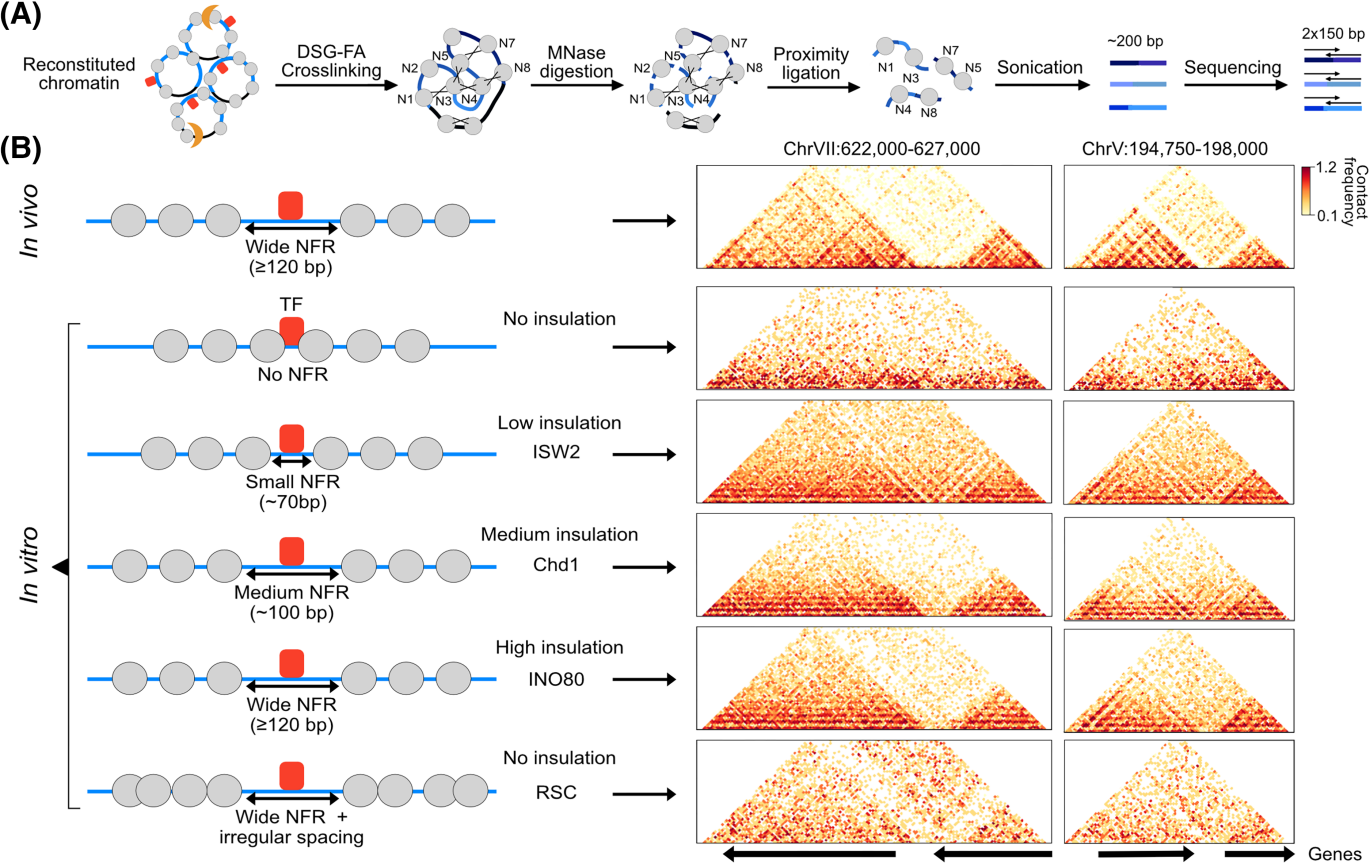
*In vitro* reconstitution of chromatin domains in *S. cerevisiae*. (**A**) Schematic overview of an *in vitro* Chromosome Conformation Capture (3C) procedure to map the 3D structure of reconstituted chromatin at sub-nucleosome resolution. Reconstituted chromatin (prepared as described in Figure 1B) is cross-linked with disuccinimidyl glutarate (DSG) and formaldehyde (FA) and digested with MNase, which is followed by proximity ligation, sonication and sequencing. (**B**) Comparison of reconstituted chromatin that has been incubated with different remodelers shows that the positioning of nucleosomes has an important role in determining higher-order chromatin structures. *In vitro* 3C data [24] for two example regions are shown, with the corresponding *in vivo* [55] data at the top for comparison.

As a proof of concept, we recently demonstrated that it is possible to reconstitute the chromatin domains that characterize *S. cerevisiae* chromosomes in interphase [24]. Using the SGD reconstitution system combined with *in vitro* remodeling reactions containing purified TFs and chromatin remodelers, we created *in vivo-*like chromatin, characterized by the stereotypical NFR-array pattern [23,39]. To map the higher-order structures of the reconstituted chromatin at sub-nucleosome resolution, we adapted high-resolution Chromosome Conformation Capture (3C) approaches [53,54], which allow for identification of the spatial organization of chromatin based on proximity ligation coupled with high-throughput sequencing (Figure 2A) [56,57]. These experiments revealed that regularly spaced nucleosome arrays that are phased to TF binding sites spontaneously fold into chromatin domains with remarkable similarity to *in vivo* domains at corresponding regions (Figure 2B) [24]. The boundaries of these domains form at NFRs at the TF binding sites and their boundary strength is dependent on the width of the NFR. Comparison of different remodelers that set distinct nucleosome linker lengths revealed that the compaction of the reconstituted domains is dependent on nucleosome linker length, with longer linkers forming more compact structures. Together, these experiments demonstrate that the positioning of nucleosomes in linear arrays impacts on the 3D configuration of chromatin. This is consistent with computer simulations that have shown that nucleosome positioning alone can predict the 3D organization of yeast interphase chromosomes [58,59].

Although these experiments were performed with yeast chromatin, they may have interesting implications for higher eukaryotes. Super-resolution microscopy studies in human and mouse cells have shown that mammalian nucleosomes are arranged in ‘clutches’ that are separated by NFRs [5] and thus bear resemblance to yeast chromatin domains. This indicates that at the smallest scale, the formation of clutches or domains across eukaryotes may be driven by the stiffness of naked DNA in NFRs that acts as a rigid spacer between neighboring regions of more flexible histone-bound DNA. This questions a direct role for transcription in the formation of chromatin domains at this scale, which has been proposed based on the observations that domain boundaries overlap with active gene promoters and that boundary strength scales with increased RNA polymerase II binding [55,60–63]. However, it is important to note that transcription and nucleosome positioning are closely connected, since active gene promoters are characterized by regular nucleosome arrays and NFRs, of which the width correlates with Pol II activity. As a result, it remains unclear whether transcription has a driving role in genome folding independent from the influence of transcription on nucleosome positioning.

## Reconstitution of loop extrusion by SMC complexes

**Figure 3.**
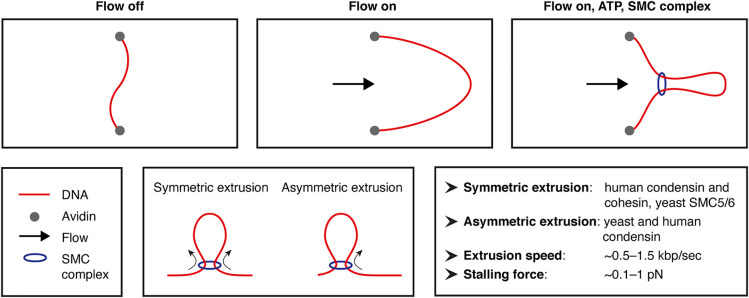
*In vitro* reconstitution of loop extrusion by SMC complexes. The top row shows a schematic overview of the experimental set-up to study loop extrusion on single DNA molecules. Biotin-labeled DNA is tethered to an avidin-coated glass slide. By applying continuous buffer flow and adding ATP and fluorescently-labeled SMC complexes, loop extrusion can be imaged in real-time. The bottom row shows a figure legend, a schematic overview of symmetric and asymmetric loop extrusion, and a brief summary of the insights that have been provided by *in vitro* single-molecule experiments with SMC complexes.

Although interphase chromatin domains in yeast can be reconstituted in absence of SMC complexes [24], *in vivo* experiments based on perturbation of SMC subunits have provided strong evidence for an important role for SMC complexes in the organization of interphase chromatin in mammals [64,65] and mitotic chromosome structures across eukaryotes [66,67]. However, due to the dynamic nature of SMC-complex-mediated loop extrusion, it has been difficult to gain insight into the molecular details of this process in *in vivo* studies. Recently, this challenge has been addressed by innovative *in vitro* experiments with purified SMC complexes in an experimental set-up in which DNA molecules are tethered at both ends to a passivated surface in a loose, low-tension configuration and stretched by buffer flow. Real-time, single-molecule imaging of the DNA molecules in presence of SMC proteins and ATP in this set-up allows for direct visualization of loop formation (Figure 3) [14]. These experiments have provided the first unambiguous evidence that *S. cerevisiae* condensin is an active motor protein that can extrude a large DNA loop of tens of kbp [68]. Subsequent experiments in a similar experimental set-up with human condensin [69] and cohesin complexes [70,71] and yeast SMC5/6 complexes [72] have demonstrated active extrusion of loops by these complexes as well, indicating that loop extrusion is likely a common mechanism of SMC complexes.

These *in vitro* single-molecule experiments have resolved important open questions concerning the molecular features of loop extrusion [13,14]. The symmetry of extrusion, i.e. whether SMC complexes reel DNA into loops from one side or both sides, has implications for the resulting extrusion patterns (Figure 3). Interestingly, yeast condensin extrudes DNA asymmetrically [68]; human condensins are capable of both symmetric and asymmetric extrusion [69]; and human cohesin and yeast SMC5/6 complexes extrude loops in a symmetric way [70–72]. There are many shared molecular features as well. All investigated SMC complexes appear to be weak motors, which stall when tension in the extruded DNA accumulates [68,70–72]. Despite their low stalling force, SMC complexes are relatively fast motors, with *in vitro* loop extrusion speeds of ∼0.5–1.5 kbp/s [68,70–72]. A recent study based on experiments with magnetic tweezers has proposed that yeast condensin achieves this by reeling in DNA with step sizes of ∼20–40 nm, corresponding to ∼200 bp [73]. On the basis of these and other biophysical, biochemical and structural experiments with SMC complexes, a mechanistic ‘reel-and-seal’ model for DNA loop extrusion has recently been proposed, which makes interesting predictions to test in further *in vitro* and *in vivo* experiments [74].

Although the above-mentioned *in vitro* studies have been instrumental for our understanding of the mechanisms by which SMC complexes organize eukaryotic genomes, an important limitation is that they are based on histone-free DNA. Interestingly, two recent studies have demonstrated that yeast and human condensins can bypass individual nucleosomes and incorporate them into extruded loops [69,75]. Although this indicates that individual nucleosomes do not form a roadblock to loop extrusion, it remains unclear to what extent the parameters derived from studies with naked DNA apply to *in vivo-*like chromatin templates. Furthermore, it is possible that the precise positioning of nucleosomes directly influences loop extrusion trajectories. Over the last years, it has become clear that specific interactions between extruding cohesin complexes and CTCF proteins lead to the formation of insulated domains (TADs) that are separated by CTCF-bound borders [76–78]. It is of interest that CTCF also has a strong ability to position nucleosomes in regular arrays [79]. Consistent with the driving role of nucleosome positioning in yeast genome organization [24], it has been shown that CTCF binding mediates local insulation in mammalian genomes independent of loop extrusion by cohesin [53]. It is conceivable that the nucleosome arrangement at CTCF binding sites also contributes to their ability to halt extruding cohesin molecules, which is consistent with a recent study that demonstrated that perturbation of nucleosome positioning at CTCF binding sites by deletion of the ISWI ATPase leads to a decrease in insulation between TADs [80]. In addition to a role for nucleosome positioning in positioning SMC complexes on chromatin, it has also been proposed that the arrangement of nucleosomes impacts on cohesin loading on chromatin and that remodeling is required for efficient recruitment of cohesin [81].

## Conclusion and outlook

The above-mentioned studies have demonstrated that *in vitro* reconstitution experiments provide a useful approach to gain insight into the molecular mechanisms underlying the 3D organization of the genome and the function of the resulting chromatin structures in modulating nuclear processes. Further development of reconstitution systems may therefore be valuable for addressing open questions in the chromatin field. For example, integrating histone modifications in reconstituted chromatin could allow for detailed investigation of the cause-consequence relationship between the ‘histone code’, chromatin organization, and transcriptional regulation [82,83]. In addition, the development of a system in which reconstitution of larger regions of chromatin [84] with *in vivo*-like nucleosome positioning is combined with single-molecule analysis of SMC complexes and/or *in vitro* transcription experiments could resolve important open questions about the interplay between nucleosome positioning, loop extrusion and transcription in the context of higher-order chromatin structures.

## Perspectives

- The 3D organization of the genome has an important role in many nuclear processes, including the regulation of transcription. Due to the complex nuclear environment, *in vitro* reconstitution approaches provide a useful tool to determine the molecular mechanisms by which chromatin structures form and function.
- Current *in vitro* reconstitution studies have contributed to resolving the mechanisms that position nucleosomes across the genome and have provided important insights into the processes that drive the 3D organization of nucleosomes into chromatin domains.
- The development of more elaborate chromatin reconstitution systems holds great potential for future investigations into the mechanistic interplay between histone modifications, chromatin remodeling, higher-order chromatin folding, and transcription.

## Open Access

Open access for this article was enabled by the participation of Max Planck Digital Library in an all-inclusive *Read & Publish* agreement with Portland Press and the Biochemical Society under a transformative agreement with MPDL.

## Abbreviations

MNase-seq: micrococcal nuclease-sequencing
NFR: nucleosome-free region
SGD: salt gradient dialysis
SMC: Structural Maintenance of Chromosomes
TAD: topologically associating domain
TF: transcription factor
TSS: transcription start site
